# Chest X-Ray as a Screening Tool for Aortic Arch Dilation: CT-Based Evaluation of Reliability

**DOI:** 10.3390/diagnostics15202564

**Published:** 2025-10-11

**Authors:** Maciej Lis, Robert Banyś, Bernard Solewski, Aleksandra Stanek, Maciej Krupiński, Barbara Obuchowicz, Tomasz Puto, Adam Piórkowski, Krzysztof Batko

**Affiliations:** 1 Clinical Imaging Diagnostics Center, 5th Military Hospital with Polyclinic in Krakow, 30-901 Krakow, Poland; 2 Department of Anatomy, Jagiellonian University Medical College, 31-008 Krakow, Poland; 3 Department of Diagnostic Imaging, Faculty of Medicine, Andrzej Frycz Modrzewski Krakow University, 30-705 Krakow, Poland; 4 Department of Conservative Dentistry with Endodontics, Jagiellonian University Collegium Medicum, Montelupich 4, 31-155 Krakow, Poland; 5 Department of Biocybernetics and Biomedical Engineering, AGH University of Krakow, 30-059 Krakow, Poland; 6 Department of Dermatology and Allergology, University Hospital, 30-668 Krakow, Poland; 7 Department of Research and Development, Medicine Economy Law Society (MELS) Foundation, 30-040 Krakow, Poland; 8 Doctoral School of Medical and Health Sciences, Jagiellonian University, 30-010 Krakow, Poland

**Keywords:** chest X-ray, aortic arch dilation, aortic knob width, computed tomography angiography, screening tool

## Abstract

**Background**: Chest radiography (CXR) remains the most common first-line imaging for thoracic abnormalities. While aortic knob width can reflect aortic dilation, no standardized, widely recognized thresholds of clinical utility exist. **Methods**: This pilot retrospective study analyzed 240 emergency department patients (median age 67 years, 61% male) who underwent both PA CXR and chest computed tomography angiography (CTA) within 7 days. Three aortic knob dimensions (horizontal, oblique, vertical) were measured on CXR and compared with CTA measurements at two anatomical levels: proximal to the brachiocephalic trunk (P-BCT) and distal to the left subclavian artery (D-LSA). **Results**: The horizontal aortic knob width was most closely related to CTA measurements of P-BCT and D-LSA. A regression model incorporating horizontal knob diameter, age, and sex was characterized with an AUC of 0.884 (95% CI 0.825–0.944) for detecting aortic dilation (>40 mm). Using a conservative threshold with the upper 95% prediction bound exceeding 40 mm led to 100% sensitivity and 54% specificity, with a negative predictive value of 1.00. **Conclusions**: Simple quantitative CXR measurements of aortic knob width (horizontal), combined with age and sex, can provide additional confidence for excluding aortic arch dilation. Given further validation in diverse populations, if the high negative predictive value of this approach will be confirmed, it may represent a valuable screening tool to guide decisions for advanced imaging, especially due to low cost and wide availability.

## 1. Introduction

Despite rapid advances in imaging, chest radiography (CXR) remains a pivotal aspect of medical diagnostics across the globe [1,2,3,4,5]. The aortic knob, a landmark on posteroanterior CXRs, is viewed as an early indicator of aortic pathology [3]. Historically, a widened aortic knob has been a subjective marker for pathological conditions like thoracic aortic aneurysms. Although some studies have explored normal dimensions [2,3,5], there is no consensus on specific CXR thresholds for further evaluation. The decision making process for more advanced follow-up imaging like CTA remains based on individual expertise of the reader [6]. At the same time, the presence of multiple imaging modalities makes development of standardized operating procedures a difficult task [6,7,8].

The subjective interpretation of the aortic knob contributes to the known limitations of CXR in diagnosing aortic disease, with studies reporting variable sensitivity and specificity [9,10,11]. Given that thoracic aortic aneurysms are often asymptomatic until they become life-threatening, early detection is crucial [12]. While CTA is the gold standard for diagnosis, it is not suitable for general population screening.

This study evaluates the clinical utility of CXR-based measurements, focusing on aortic knob width in different orientations. We compared CXR parameters with CTA-based aortic arch dimensions to evaluate potential diagnostic performance and establish clinical relevance as a screening tool for aortic dilation.

## 2. Materials and Methods

This study was conducted in accordance with the ethical principles outlined in the Declaration of Helsinki. The research protocol received approval from the OIL Chamber of Physicians Ethical Council in Krakow (Approval No. 53/KBL/OIL/2025 from 15 July 2025).

### 2.1. Population

A retrospective search was conducted using the institutional PACS database to identify emergency department (ED) patients who had undergone both PA CXR and chest CTA within a 7-day interval. Exclusion criteria included: patient age < 18 years, inadequate chest CTA image quality (due to low contrast enhancement or motion artifacts), chest wall deformities, significant scoliosis, history of prior aortic surgery or endovascular repair, and acute aortic syndromes.

### 2.2. Measurement and Definitions

Two thoracic aortic levels were selected for CTA analysis: (1) the ascending aorta just proximal to the origin of the brachiocephalic trunk (P-BCT level) and (2) the aortic arch just distal to the origin of the left subclavian artery (D-LSA level). These segments represent the regions of the aortic arch that form the aortic knob silhouette on chest radiographs.

**Figure 1:**
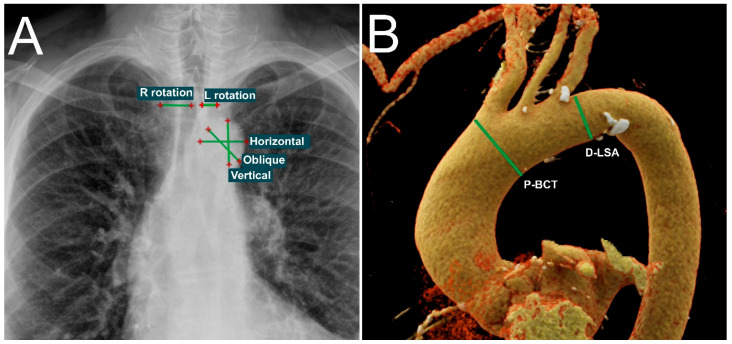
Measurement methodology. (**A**) Posteroanterior chest radiograph with overlaid annotations illustrating key parameters. R-rotation and L-rotation indicate the distances from each clavicle to the nearest spinous process, used to assess patient rotation. Horizontal, oblique, and vertical lines represent the three aortic knob measurements analyzed. (**B**) Volumetric CTA reconstruction focused on the thoracic aorta. Green lines indicate anatomical measurement levels: P-BCT (proximal to the origin of the brachiocephalic trunk) and D-LSA (distal to the origin of the left subclavian artery).

On CXR, the aortic knob was measured in three dimensions (see Figure 1):

- The horizontal diameter was defined as the maximum distance between the left border of the trachea and the most lateral projection of the aortic knob.
- The oblique diameter was measured as the longest distance between any two visible borders of the knob.
- The vertical diameter extended from the uppermost margin of the knob to the lowest visible shadow indicating the downward bend of the aortic arch.

Additional radiographic parameters included patient rotation, assessed by comparing the distance between each sternal end of clavicle and the closest spinous process; chest width, measured at the level of the diaphragm; and the presence of aortic arch calcifications, evaluated visually. On CTAs, we further assessed the span of the aortic arch by measuring the longest horizontal and vertical distances between visible aortic segments at the axial slice corresponding to the bifurcation of the pulmonary trunk. Calcifications of the arch were also recorded.

All image measurements were independently performed by three radiologists with expertise in interpreting chest radiographs and CT angiography. CXR measurements were conducted using RadiAnt DICOM Viewer (Medixant, Poznań, Poland), while CTA measurements were carried out using syngo.via software version VB80 (Siemens Healthineers, Erlangen, Germany). Representative examples of the measurement methodology are shown in Figure 1.

### 2.3. Statistical Analysis

Statistical analyses were conducted using R version 4.4.1 (R Core Team, 2024; R Foundation for Statistical Computing, Vienna, Austria). Nominal variables were presented as counts and percentages (N, %). The distribution of continuous variables was assessed through density plots, the Shapiro-Wilk & Anderson-Darling tests, with supplemental measures of skewness. Continuous data were summarized as either mean (standard deviation) or median (interquartile range), as deemed appropriate. Robust regression models were developed using generalized additive models (GAMs using the mgcv package). Variable selection was guided by a combination of domain expertise and machine learning techniques. Random forest (RF) models were constructed using default settings to minimize overfitting, given sample size constraints and the avoidance of split-sample tuning. A parsimonious set of candidate predictors from 2D chest X-ray measurements was derived based on joint team discussions and the analysis of global variable importance plots.

No a priori sample size calculation was performed for this exploratory study. We provide confidence intervals and acknowledge that our findings require external validation. For tree-based predictive models or variable selection, no standardized methods for sample size calculation are available. These algorithms benefit from larger sample size and strong predictor variables, but multiple dataset characteristics can affect performance [13].

### 2.4. Modeling Approach

We employed GAMs to analyze the relationship between chest radiography and CT measurements. These models are flexible enough to capture both linear and non-linear relationships in the data. Prior to GAM modeling, we utilized random forest (an aggregate decision tree model) to identify the most important predictive model features in a robust manner. Random forest algorithms enable complex, nonlinear modeling of relationships and are established as best-in-class tools for tabular data of moderate size [14,15]. Model optimization was performed using internal cross-validation techniques. To maximize interpretability, we report R^2^ as the primary measure of model fit.

## 3. Results

### 3.1. Characteristics of the Derivation Cohort

A total of 240 consecutive patients who underwent both posteroanterior chest radiography and chest CT angiography within a 7-day interval were included in this analysis. Complete case analysis was performed. Basic demographic and detailed imaging characteristics are presented in Table A1 (see Appendix A). The study population showed a wide age range, with both young adults and elderly individuals included. Modest male predominance was noted. Chest radiography parameters suggest good positioning, with minimal rotation differences (<5 mm between left and right clavicles), across the cohort.

Aortic knob morphometric characteristics reflect common anatomical patterns. The horizontal dimension was consistently the largest measurable aortic knob dimension, followed by oblique and vertical measurements.

Comparison of CT assessment showed superior detection of calcification over conventional radiography. Angiographic measurements of the aorta at the two predefined anatomical levels are in line with the expected anatomical structure of the aortic arch. Specifically, larger diameters are observed at the P-BCT level when comparing to the D-LSA level.

Inter- and intra-observer agreement analysis suggests high reproducibility for all measurement parameters. Details in Table A2 (see Appendix A).

### 3.2. Relationships Between CXR and CT Measurements of the Aorta

**Figure 2:**
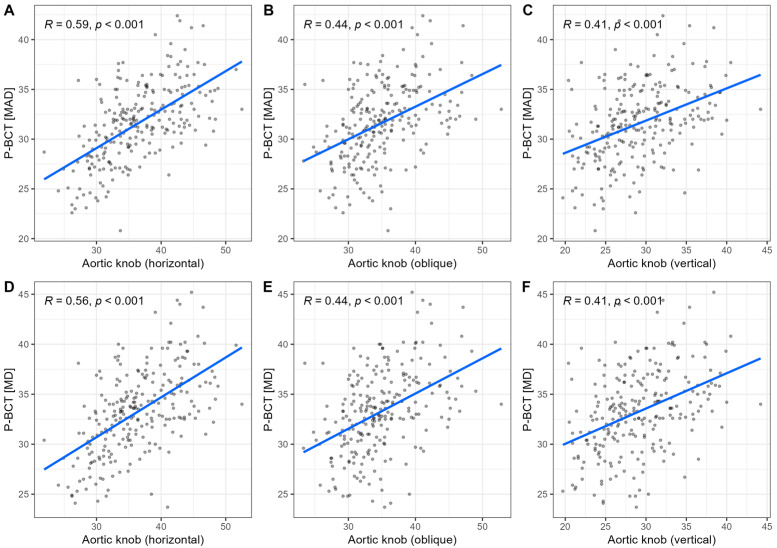
Linear relationship between aortic knob measurements (**A**,**D**—horizontal, **B**,**E**—oblique, **C**,**F**—vertical) and aortic dimensions (**A**–**C** for MAD; **D**–**F** for MD) from computed tomography angiography.

We examined linear relationships across both CT and CXR measurements using Pearson’s correlation analysis (see Figure 2). Among the aortic knob dimensions, the horizontal diameter exhibited a strong positive correlation with the oblique diameter (r = 0.72), and a moderate correlation with the vertical dimension (r = 0.55). Similarly, the oblique and vertical diameters were closely related (r = 0.68), reflecting consistent morphological relationships within the aortic contour. Transverse chest width showed only weak positive correlations with the horizontal (r = 0.27), oblique (r = 0.34), and vertical (r = 0.26) aortic knob measurements, suggesting that overall thoracic size had limited influence on aortic knob morphology.

Both the P-BCT MAD and MD (r = 0.95), as well as the D-LSA MAD and MD (r = 0.98) showed very strong correlations, indicating high consistency between both methods of aortic assessment.

The aortic arch dimension ratio (AP/RL) showed little to no association with two-dimensional aortic knob measures (r = −0.17 for horizontal; r = −0.07 for oblique; r = −0.01 for vertical). The linear relationship with CT-based aortic measurements was also modest; P-BCT MAD and MD (r = −0.18, −0.18, respectively) and D-LSA MAD and MD (r = −0.19, −0.18, respectively).

### 3.3. Modeling of the Aortic Arch Dimensions Based on Chest X-Ray Data

#### 3.3.1. Variable Selection Using Random Forest

**Figure 3:**
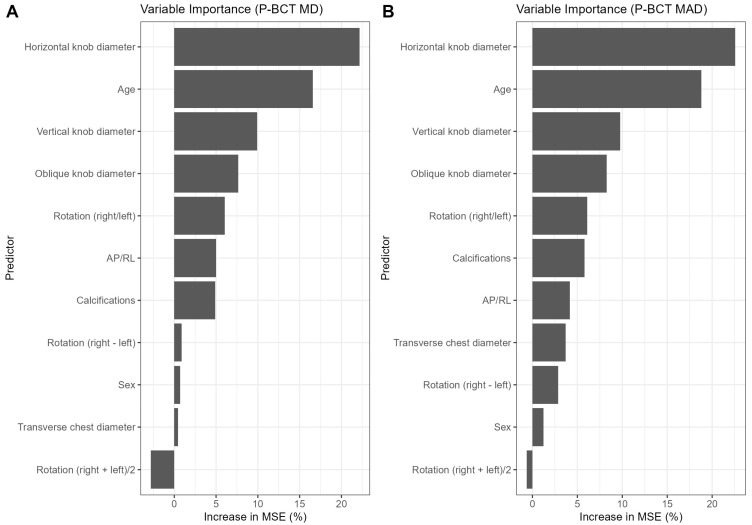
Feature importance for predicting CT-based aortic dimensions at the P-BCT level (**A**—MD, **B**—MAD) based on random forest models.

We evaluated a pre-specified set of CXR variables with random-forest models for both CT-based aortic measurements at the P-BCT level. Candidate variables were defined based on technical considerations and study team discussions. Potential predictors were age, sex, horizontal/oblique/vertical aortic knob diameters, transverse chest width, three rotation-correction measures and presence of CXR calcifications. Due to the moderate sample size and bias risk, we did not derive additional combinations and focused on simple measurements that are likely to be reproducible. Based on feature importance (see Figure 3—Panel A and B) we identified the horizontal knob dimension and age as the most useful parameters. Vertical and oblique measures appeared to be less effective predictive features, while other potentially relevant covariates were of small or negligible value.

#### 3.3.2. Comparison of Generalized Additive Models with Age, Sex and Rotational Adjustment

We fitted GAMs for each radiographic aortic knob measure (horizontal, oblique, vertical) aiming to predict CT-based diameters at the P-BCT level. Firstly, we considered univariable (i.e., each aortic knob dimension alone) models, which were extended with age and sex addition, and finally, a rotation term in the last step. All smooth terms were significant (*p* < 0.001; Table A3). Likelihood-ratio deviance tests suggested the horizontal dimension as best model for both P-BCT measurements (MAD *p* < 0.001; MD *p* = 0.004).

In age- and sex-adjusted models (Table 1), the horizontal aortic knob diameter explained the most variance for both outcomes (R^2^ = 0.412 for MAD; 0.365 for MD), which is superior to the oblique (R^2^ = 0.355; R^2^ = 0.321, respectively) and vertical (R^2^ = 0.328; R^2^ = 0.297, respectively) models. Adding age and sex improved model fit relative to the crude models for all predictors. In contrast, the rotational correction term produced minimal changes in fit (change in adj. R^2^ below +0.010 across modeling structures; moreover, it was statistically non-significant for both P-BCT measures).

Taking the above into account, the horizontal aortic diameter was selected as the final model structure.

#### 3.3.3. Establishing the Functional Form and Final Equation

Aortic diameter was defined as the maximum dimension from either MD or MAD assessment. Following feature selection (as described above), age, sex, and horizontal aortic knob diameter were identified as the optimal parsimonious model feature set. Given nonlinearity in relationships (see Table 1), we considered different modeling options. Linear regression was selected over more complex GAM modeling due to comparable performance (e.g., R^2^ 0.405, R^2^ 0.364 for P-BCT MAD and MD model). The chosen model incorporated square root transformation of horizontal aortic diameter with additive terms for age and sex, which was guided by theoretical considerations reflecting the allometric scaling relationship [16,17]. Final model performance was estimated using AUC 0.884 (95% CI 0.825–0.944), which indicates high predictive potential. This model was utilized to create reference tables based on the prediction interval (see Table A3 and Table A4).

For comparative purposes, we considered different cut-offs. The Youden-derived cut-off for the predicted diameter was 35.84 mm, providing 91% sensitivity and 84% specificity, with modest PPV (0.213) and high NPV (0.995). A potential, crude pragmatic cut-offs of 35 mm would yield 91% sensitivity, 73% specificity, PPV of 0.137 and NPV of 0.994. In turn, increasing the threshold to 36 mm would reduce sensitivity to 46% while improving specificity to 86%, with PPV at 0.132 and NPV of 0.970.

**Figure A1:**
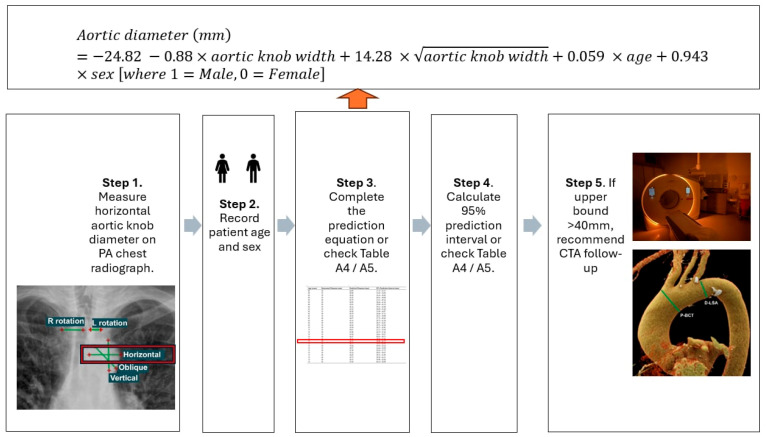
Proposed flowchart of clinical decisions for integration into chest X ray protocols.

For pilot clinical application (overview in Figure A1), we propose a conservative rule: flagging dilation and recommending follow-up CTA when the upper 95% prediction bound exceeds 40 mm. In our cohort, this approach captured all cases of dilation (sensitivity 100%) with 54% specificity, a PPV of 0.093, and an NPV of 1.000.

## 4. Discussion

This study demonstrates that simple, quantitative measurements of the aortic knob on routine posteroanterior chest radiographs can provide clinically useful insights. The salient findings of this report are: (1) CT-based aortic arch dimensions are moderately correlated with various measurements of aortic knob width on CXR; (2) the horizontal dimension is most closely related to P-BCT and D-LSA dimensions; (3) combining CXR measures with demographic information into a simple linear regression equation with square root transformation provides a readily accessible tool with high negative predictive value, pending further validation. If similar results are reported in various clinical populations, CXR assessments will be able to provide additional clinical reassurance and guide more advanced imaging decision making with high confidence.

When examining the three CXR dimensions of the aortic knob (horizontal, vertical, and oblique assessments), we observed that all measurements showed moderate strength inter-relationships with CT-based aortic parameters. However, the horizontal knob width was consistently observed to be most closely related to 3D dimensions. Our observations extend the findings of prior studies, in which the horizontal (transverse) or left mediastinal width was described as a useful and reliable measurement, contrasting with other CXR-based parameters or orientations [3,18]. Moderate-to-strong correlations between aortic knob measurements across different radiographic projections are likely to reflect three-dimensional aortic arch geometry. The square root transformation was also selected because aortic dimensions scale allometrically with body surface area. In contrast, weak correlations between transverse chest width and knob measurements suggest that aortic knob size is independent of overall thoracic dimensions and reflects intrinsic vascular geometry.

A major finding of our report is that the final model with age and sex-adjustment and square root transformation of horizontal knob width enables calculation of a prediction threshold that successfully identifies all cases of CT-detected aortic dilatation. We favored a simple model over the more mathematically complex GAM due to several theoretical considerations [16,17]. It should be emphasized that there was a low prevalence of dilation in our cohort, which necessitates caution (low pre-test probability) and reflects our approach to favor a very conservative rule, wherein sensitivity is prioritized over specificity. We also observed that inter- and intra-rater reliability for radiographic knob measurements, as well as CT-based aortic assessments was very high, which strengthens the confidence in reproducibility and clinical implications of this pilot study. Our findings support future efforts to incorporate standardized aortic knob assessment as an inherent part of routine CXR description protocols, particularly in older adults or patients with CV risk factors.

Our study focused on the segment of the thoracic aorta corresponding anatomically with the aortic knob silhouette; the distal ascending aorta and the transverse arch (i.e., between the origin of the P-BCT and D-LSA). We excluded aneurysms of the descending thoracic aorta from analysis, as they are much more likely to affect aortic shadow than just the knob itself. By limiting CT evaluations to these arch-specific region, we are likely to more confidently attribute knob enlargement on CXR to dilation of the arch, rather than adjacent structures. However, it should be kept in mind that aneurysms confined to the ascending aorta proximal to the brachiocephalic trunk may remain radiographically occult [11,19], as this portion usually does not significantly influence knob contour. At the same time, the prevalence of ascending aortic aneurysms is high [20,21,22,23].

Beyond age and sex considerations [23], data from literature indicate that aortic knob width on CXR is associated with the presence of CV risk factors and overt CV disease. Therefore, when interpreting aortic knob measures, it is important to consider the clinical context. For example, in hypertensive populations, a prominent aortic knob is a common finding. Rayner et al. observed that patients with long-standing hypertension have significantly greater aortic knob widths on average (~3.7 cm), when compared to normotensive individuals (~3.3 cm) [2]. Prior studies demonstrate associations between aortic knob width and hypertension-related markers of organ damage [2], aortic pulse pressure [24] as well as left ventricle diastolic function [25]. Moreover, relationships with nontraditional CV risk factors, such as obstructive sleep apnea, should be considered [26]. Due to these factors, the potential for selection bias represents a significant limitation of this report and our results require validation in heterogeneous clinical populations.

CXR based measurements of aortic dimensions have been reportd as useful in predicting aortic dissection [18]. More recent studies incorporating machine-learning tools, such as neural networks, report on high detection accuracy of aortic dissection based on CXR data [27]. However, the diagnostic limitations of CXR in detecting thoracic aortic aneurysms need to be emphasized [9,28]. Prior studies on CXR report sensitivity of 67% for overt dissection and 61% for non-dissecting aneurysm [11]. Substantial overlap in knob dimensions between normal and aneurysmal cases remains a relevant risk. CXR cannot be used as a confirmatory tool but should be viewed as an initial screening test with moderate-to-high NPV value. Real-life cases emphasize the importance of maintaining a high index of suspicion when examining CXRs; even in younger individuals without overt risk factors, as aortic dissection may occur in persons of all ages, with identifiable early signs on CXR [29].

With the ongoing search for CV biomarkers [30], investigators have linked increased knob width to markers of atherosclerosis [31,32]. Some authors have described its adjunct value for predicting mortality in special populations, such as hemodialysis patients [33]. Lee et al. demonstrated that aortic knob enlargement is independently associated with metabolic syndrome, implicating it as a surrogate for systemic atherosclerotic burden [34]. Similarly, in patients with essential hypertension, a larger aortic knob correlates with greater carotid intima-media thickness, an ultrasound measure of arterial stiffness and atherosclerosis [35]. These findings support the concept that an enlarged aortic knob on chest radiography represents not only an anatomic variant but also a potential indicator of broader CV risk.

## 5. Conclusions

This pilot study suggests that quantitative analysis of the aortic knob on routine chest radiographs is clinically valuable. We found that the horizontal aortic knob diameter was characterized by the strongest and most consistent relationship with CT-based aortic dimensions. A simple model with age and sex and square root transformation of radiographic data could be utilized to calculate a risk score with high negative predictive value for aortic dilation. Our findings support incorporating standardized aortic knob assessment into institutional protocols. Given further validation, this approach to chest X-ray interpretation may lead to the development of a validated, low-cost and widely accessible tool. Patient demographics that may benefit are older adults and patients with cardiovascular risk factors, though future validation in these populations is necessary.

## Figures and Tables

**Table 1:** Performance of radiographic predictors for CT-based aortic parameters at P-BCT level.

Radiographic Predictor	Adj. R^2^ [MD]	Change vs. Crude Model	Change with Rotation	*p* Value for Rotation	Adj. R^2^ [MAD]	Change vs. Crude Model	Change with Rotation	*p* Value for Rotation
Horizontal	0.365	+0.035	+0.000	0.351	0.412	+0.046	+0.003	0.151
Oblique	0.321	+0.119	+0.005	0.354	0.355	+0.145	+0.004	0.097
Vertical	0.297	+0.132	+0.010	0.202	0.328	+0.163	+0.008	0.051

Notes: R^2^ reflects the proportion of variance in CT parameters explained by each predictor after adjustment for age and sex. The comparison with the crude (univariable model) indicates improvements in performance, while the change with rotation column reflects potential effects of including clavicular rotation within the model.

## Data Availability

Data are available upon reasonable request.

## Abbreviations

AP	Anteroposterior
AUC	Area under the curve
CXR	Chest X-ray
CTA	Computed tomography angiography
CT	Computed tomography
CV	Cardiovascular
D-LSA	Distal to left subclavian artery
EDF	Effective degrees of freedom
ED	Emergency department
GAM	Generalized additive model
GCV	Generalized cross-validation
ICC	Intraclass correlation coefficient
IQR	Interquartile range
MAD	Mean aortic diameter (effective diameter)
MD	Maximum diameter
NPV	Negative predictive value
PA	Posteroanterior
P-BCT	Proximal to brachiocephalic trunk
PACS	Picture archiving and communication system
PPV	Positive predictive value
RF	Random forest
SD	Standard deviation
UJCM	Jagiellonian University Medical College

## Appendix A

**Table A1:** Clinical and Imaging Characteristics of the Study Cohort.

Parameter	Descriptive Statistics
Patient Demographics	
Age, median (IQR), years	67 (56–76)
Male sex, N (%)	147 (61)
Chest Radiograph Measurements	
Rotation assessment	
Left rotation, mean (SD), mm	16.30 (9.62)
Right rotation, mean (SD), mm	15.40 (9.49)
Transverse chest dimension, median (IQR), mm	308 (284–333)
Calcifications visible, N (%)	99 (41)
Aortic Knob Measurements	
Horizontal diameter	
Median (IQR), mm	35.8 (32.0–41.0)
Mean (SD), mm	36.4 (5.87)
Oblique diameter	
Median (IQR), mm	34.0 (31.2–38.2)
Mean (SD), mm	34.8 (5.23)
Vertical diameter	
Median (IQR), mm	28.6 (25.6–32.1)
Mean (SD), mm	29.1 (4.83)
CT Measurements	
Calcifications visible, n (%)	180 (75)
P-BCT Level	
MAD, median (IQR), mm	31.6 (29.2–34.2)
MAD, mean (SD), mm	31.6 (3.85)
MD, median (IQR), mm	33.3 (30.5–36.0)
MD, mean (SD), mm	33.3 (4.16)
D-LSA Level	
MAD, median (IQR), mm	26.2 (23.9–28.4)
MAD, mean (SD), mm	26.3 (3.18)
MD, median (IQR), mm	27.6 (25.7–30.2)
MD, mean (SD), mm	27.8 (3.37)
Aorta at Pulmonary Trunk Bifurcation	
Anterior–posterior dimension, mean (SD), mm	109.0 (19.3)
Right-lateral dimension, mean (SD), mm	60.8 (16.1)

Abbreviations: IQR, interquartile range; SD, standard deviation; MAD, mean area diameter; MD, maximum diameter; P-BCT, proximal to brachiocephalic trunk; D-LSA, distal to left subclavian artery.

**Table A2:** Reliability metrics for rater evaluation.

Variable	Intra-Rater ICC (95% CI, *N* = 119)	Inter-Rater ICC (95% CI, 3 Raters, *N* = 18)
Aorta pre-BCT MAD	0.90 (0.86–0.93)	0.954 (0.900–0.981)
Aorta pre-BCT MD	0.92 (0.89–0.94)	0.900 (0.793–0.959)
Aorta D-LSA MAD	0.91 (0.88–0.94)	0.923 (0.837–0.969)
Aorta D-LSA MD	0.91 (0.87–0.94)	0.941 (0.874–0.976)
Aortic knob horizontal	0.89 (0.85–0.92)	0.934 (0.858–0.973)
Aortic knob oblique	0.78 (0.69–0.84)	0.637 (0.373–0.833)
Aortic knob vertical	0.71 (0.61–0.79)	0.611 (0.340–0.819)

Abbreviations: P-BCT, Proximal to brachiocephalic trunk; D-LSA, Distal to left subclavian artery; ICC, intraclass correlation coefficient.

**Table A3:** Comparison of crude and extended generalized additive models evaluating the association between candidate aortic knob dimensions on CXR in predicting CT-based aortic diameters measured at the P-BCT level.

CT-Based	Model Construct	CXR Predictor	EDF	F (Smooth)	*p* Value	Adj. R^2^	Deviance Explained (%)	GCV	Rotation *p* Value
P-BCT MD	Univariate	Horizontal diameter	2.266	40.74	<0.001	0.330	33.7	11.736	
	Univariate	Oblique diameter	2.876	16.84	<0.001	0.202	21.2	14.017	
	Univariate	Vertical diameter	1.000	48.09	<0.001	0.165	16.8	14.559	
	Age + Sex	Horizontal + Age + Sex	1.955	19.60	<0.001	0.365	37.9	11.252	
	Age + Sex	Oblique + Age + sex	2.841	8.55	<0.001	0.321	33.8	12.085	
	Age + Sex	Vertical + Age + Sex	1.000	21.02	<0.001	0.297	31.0	12.427	
	Age + Sex + Rotation	Horizontal + Age + Sex + Rotation	1.962	18.75	<0.001	0.365	38.1	11.306	0.351
	Age + Sex + Rotation	Oblique + Age + Sex + Rotation	2.837	8.17	<0.001	0.326	34.8	12.096	0.354
	Age + Sex + Rotation	Vertical + Age + Sex + Rotation	1.000	21.09	<0.001	0.307	32.5	12.364	0.202
P-BCT MAD	Univariate	Horizontal diameter	2.381	45.47	<0.001	0.366	37.2	9.539	
	Univariate	Oblique diameter	3.167	16.06	<0.001	0.210	22.0	11.926	
	Univariate	Vertical diameter	1.539	23.77	<0.001	0.165	17.1	12.515	
	Age + Sex	Horizontal + Age + Sex	2.177	20.13	<0.001	0.412	42.6	8.967	
	Age + Sex	Oblique + Age + sex	3.123	7.78	<0.001	0.355	37.2	9.877	
	Age + Sex	Vertical + Age + Sex	1.000	19.12	<0.001	0.328	34.2	10.220	
	Age + Sex + Rotation	Horizontal + Age + Sex + Rotation	2.152	19.41	<0.001	0.415	43.1	8.964	0.151
	Age + Sex + Rotation	Oblique + Age + Sex + Rotation	3.059	7.49	<0.001	0.359	37.9	9.849	0.097
	Age + Sex + Rotation	Vertical + Age + Sex + Rotation	1.000	18.79	<0.001	0.336	35.2	10.143	0.051

Notes: EDF (effective degrees of freedom) is an estimate of the complexity of the relationship (i.e., the fitted smooth). For example, EDF = 1 suggests a linear relationship, while higher values imply curvature. F (smooth) and P values provide information on the significance of each predictor in the tested model. Adjusted R^2^ and deviance explained are measures of model fit, reflecting how well the predictors account for variability in the outcome. Generalized cross-validation (GCV) provides an additional criterion for model selection, where lower values indicate better expected predictive performance. In our analyses, all candidate aortic predictors were significant terms (*p* < 0.001), with the horizontal aortic measure characterized with the most variance explained. Adjustment for age and sex in modeling structure improved fit, while rotational correction generally added no meaningful improvement.

**Table A4:** Reference sheet for predicted CT aortic diameter dimensions based on CXR aortic knob width (horizontal) at specific ages (FEMALES).

Age (Years)	Horizontal Diameter (mm)	Predicted Diameter (mm)	95% Prediction Interval (mm)
60	20	24.95	17.73–32.17
60	25	28.08	21.60–34.56
60	30	30.49	24.16–36.82
60	35	32.35	26.01–38.69
60	40	33.78	27.41–40.14
60	45	34.85	28.44–41.26
60	50	35.62	29.06–42.19
60	55	36.14	29.21–43.08
65	20	25.25	18.00–32.49
65	25	28.38	21.89–34.87
65	30	30.79	24.46–37.11
65	35	32.65	26.31–38.98
65	40	34.07	27.72–40.42
65	45	35.14	28.75–41.54
65	50	35.92	29.37–42.47
65	55	36.44	29.52–43.36
70	20	25.54	18.27–32.81
70	25	28.67	22.17–35.18
70	30	31.08	24.75–37.41
70	35	32.94	26.61–39.27
70	40	34.37	28.02–40.71
70	45	35.44	29.06–41.82
70	50	36.21	29.68–42.75
70	55	36.73	29.82–43.65
75	20	25.84	18.53–33.14
75	25	28.97	22.44–35.50
75	30	31.38	25.03–37.72
75	35	33.24	26.91–39.57
75	40	34.66	28.32–41.00
75	45	35.73	29.36–42.11
75	50	36.51	29.98–43.04
75	55	37.03	30.13–43.93

Notes: Predictions based on linear regression model including horizontal aortic knob width, age, and sex. The 95% prediction intervals account for model uncertainty and individual variation. Horizontal aortic knob width measured on posteroanterior chest radiographs at widest point. All measurements in millimeters. Applies to female patients aged 60–75 years.

**Table A5:** Reference sheet for predicted CT aortic diameter dimensions based on CXR aortic knob width (horizontal) at specific ages (MALES).

Age (Years)	Horizontal Diameter (mm)	Predicted Diameter (mm)	95% Prediction Interval (mm)
60	20	25.89	18.59–33.20
60	25	29.03	22.50–35.55
60	30	31.43	25.10–37.77
60	35	33.29	26.98–39.61
60	40	34.72	28.40–41.04
60	45	35.79	29.44–42.14
60	50	36.57	30.07–43.06
60	55	37.09	30.22–43.95
65	20	26.19	18.85–33.53
65	25	29.32	22.77–35.87
65	30	31.73	25.38–38.07
65	35	33.59	27.27–39.91
65	40	35.01	28.70–41.33
65	45	36.09	29.75–42.43
65	50	36.86	30.38–43.34
65	55	37.38	30.53–44.24
70	20	26.49	19.11–33.86
70	25	29.62	23.05–36.19
70	30	32.02	25.67–38.38
70	35	33.88	27.56–40.21
70	40	35.31	29.00–41.62
70	45	36.38	30.04–42.72
70	50	37.16	30.68–43.63
70	55	37.68	30.83–44.53
75	20	26.78	19.37–34.19
75	25	29.91	23.31–36.51
75	30	32.32	25.94–38.69
75	35	34.18	27.85–40.51
75	40	35.60	29.29–41.92
75	45	36.68	30.34–43.01
75	50	37.45	30.97–43.93
75	55	37.97	31.12–44.82

Notes: Predictions based on linear regression model including horizontal aortic knob width, age, and sex. The 95% prediction intervals account for model uncertainty and individual variation. Horizontal aortic knob width measured on posteroanterior chest radiographs at widest point. All measurements in millimeters. Applies to male patients aged 60–75 years.
